# Effects of Co-existing Heterotrophs on Physiology of and Nitrogen Metabolism in Autotrophic Nitrite-oxidizing *Candidatus* Nitrotoga

**DOI:** 10.1264/jsme2.ME23076

**Published:** 2023-12-09

**Authors:** Hiroto Ide, Kento Ishii, Yu Takahashi, Hirotsugu Fujitani, Satoshi Tsuneda

**Affiliations:** 1 Department of Life Science and Medical Bioscience, Waseda University, Tokyo, Japan; 2 Research Organization for Nano & Life Innovation, Waseda University, Tokyo, Japan; 3 Department of Biological Sciences, Chuo University, Tokyo, Japan

**Keywords:** co-culture, growth, interaction, nitrite-oxidizing bacteria, transcriptome

## Abstract

Interactions between autotrophic nitrifiers and heterotrophs have attracted considerable attention in microbial ecology. However, the mechanisms by which heterotrophs affect the physiological activity of and nitrogen metabolism in autotrophic nitrite oxidizers remain unclear. We herein focused on nitrite-oxidizing *Candidatus* Nitrotoga and compared an axenic culture including only *Ca.* Nitrotoga with a co-culture of both *Ca.* Nitrotoga and *Acidovorax* in physiological experiments and transcriptomics. In the co-culture with *Acidovorax*, nitrite consumption by *Ca.* Nitrotoga was promoted, and some genes relevant to nitrogen metabolism in *Ca.* Nitrotoga were highly expressed. These results provide insights into the mechanisms by which co-existing heterotrophs affect autotrophic nitrifiers.

Conventional nitrification is a two-step reaction: ammonia oxidation catalyzed by ammonia-oxidizing archaea (AOA) and bacteria (AOB) followed by nitrite oxidation catalyzed by nitrite-oxidizing bacteria (NOB). Recent studies reported that some members of the genus *Nitrospira* completely oxidize ammonia to nitrate via nitrite in a single cell (Daims *et al.*, 2015; van Kessel *et al.*, 2015). These nitrifiers have been recognized as chemolithoautotrophs that utilize ammonia or nitrite as an energy source and fix carbon dioxide as a carbon source. A more detailed understanding of the interactions between autotrophic nitrifiers and heterotrophs is needed from the perspective of biogeochemical nitrogen cycles and artificial water treatment processes. However, one of the largest obstacles to addressing these research issues is the difficulties associated with the cultivation and isolation of nitrifiers. Therefore, mole­cular and physiological approaches with environmental samples, including nitrifier biofilms, are useful for investigating interactions between nitrifiers and heterotrophs. For example, microautoradiography (MAR) in combination with fluorescence *in situ* hybridization (FISH) revealed that some nitrifiers assist the growth of heterotrophs by supplying secondary metabolites in autotrophic nitrifying biofilms (Kindaichi *et al.*, 2004; Okabe *et al.*, 2005). Multiple approaches combining mole­cular techniques, microelectrode measurements, and mathematical modeling have indicated that metabolites produced by autotrophic nitrifiers affect microbial community structures in nitrifying granules (Matsumoto *et al.*, 2010). These findings demonstrate that heterotrophs benefit from co-existing nitrifiers, which may be indispensable for survival in oligotrophic environments.

The number of phylogenetically novel nitrifier isolates has steadily increased with the development of state-of-the-art cultivation methods and continuous efforts by researchers (Koch *et al.*, 2019). With increases in the availability of pure cultures of nitrifiers, attention is now focused on whether autotrophic nitrifiers receive any benefits from co-‍existing heterotrophs. A combination of physiological experiments and proteomics revealed that the growth rate of *Nitrosomonas* sp. Is79, a representative strain of AOB, increased in co-cultures with diverse heterotrophs (Sedlacek *et al.*, 2016). Furthermore, heterotrophs growing with meta­bolites of nitrite-oxidizing *Nitrospira* provided growth-promoting factors to *Nitrospira*, which appeared to be a mutualistic relationship between autotrophic NOB and heterotrophs (Murakami *et al.*, 2022). Based on these findings, we focused on the NOB genus *Candidatus* Nitrotoga. This functional guild was initially discovered in permafrost soils in the Siberian Arctic and was described as *Ca.* Nitrotoga *arctica* (Alawi *et al.*, 2007). To date, several enrichments (Hüpeden *et al.*, 2016; Ishii *et al.*, 2017; Boddicker and Mosier, 2018; Wegen *et al.*, 2019; Lantz *et al.*, 2021; Keuter *et al.*, 2022) and two isolates (Kitzinger *et al.*, 2018; Ishii *et al.*, 2020) affiliated with the genus *Ca.* Nitrotoga have been cultured in various environments. The scarcity of cultured strains indicates that members of the genus *Ca.* Nitrotoga are fastidious and recalcitrant to laboratory conditions. Therefore, difficulties are associated with isolating *Ca.* Nitrotoga strains from mixed co-cultures with heterotrophs. Moreover, a recent review reported that *Ca.* Nitrotoga also benefits from heterotrophs (Spieck *et al.*, 2021).

To test this hypothesis, we investigated the mechanisms by which heterotrophs affect the nitrite oxidation activity and/or growth activity of *Ca.* Nitrotoga. We previously isolated the novel *Ca.* Nitrotoga sp. strain AM1P from an enrichment culture containing the genus *Acidovorax* as the most abundant contaminant (Ishii *et al.*, 2020). Physiological experiments revealed that the nitrite oxidation activity of AM1P was enhanced by ammonium, pyruvate, and catalase (Ishii *et al.*, 2020). Since members of the genus *Acidovorax* are considered to be key heterotrophs, the whole genome of *Acidovorax* sp. NB1 isolated from the enrichment culture was sequenced (Ide *et al.*, 2019). In the present study, we performed a comparative physiological test on an axenic culture of AM1P and co-culture of AM1P+NB1 and used transcriptomics to elucidate the mechanism by which NB1 affects the metabolism of AM1P.

We investigated nitrite consumption by AM1P at low cell densities (axenic culture vs. co-culture). AM1P cells were prepared under nitrite starvation for 3 days (see Supplementary Information). In 96-well microtiter plates, 150‍ ‍μL of inorganic medium containing 2.1‍ ‍mM nitrite was added to 6 wells. In three microtiter plates of the axenic culture (only AM1P), cell suspensions of AM1P were inoculated at an initial cell density of 10^2^, 10^3^, or 10^4^‍ ‍cells‍ ‍mL^–1^. In the other three microtiter plates of the co-culture (AM1P+NB1), equivalent cell suspensions of AM1P and NB1 were inoculated at 10^2^, 10^3^, or 10^4^‍ ‍cells‍ ‍mL^–1^ for each. All microtiter plates were statically incubated at 23°C in the dark. Nitrite concentrations were measured using Griess reagent on days 20 and 46 from the start of cultivation. The co-culture consumed more nitrite than the axenic culture at an initial cell density of 10^2^‍ ‍cells‍ ‍mL^–1^ on days 20 and 46 (Fig. 1A). Similar results were obtained for the initial cell densities of 10^3^ and 10^4^‍ ‍cells‍ ‍mL^–1^. To further investigate this phenomena, the timing of the initiation of nitrite consumption in all wells was estimated. We defined 0.21‍ ‍mM of nitrite, which was equivalent to 10% of the initial nitrite concentration, as the threshold for nitrite consumption. The number of wells in which nitrite consumption exceeded the threshold was counted. On day 20, the co-culture had more wells exceeding the threshold than the axenic culture at any initial cell density (10^2^–10^4^‍ ‍cells‍ ‍mL^–1^) (Fig. S1). Therefore, nitrite consumption by AM1P was promoted in the presence of NB1 during the initial incubation phase. Note that NB1 employs two approaches for the consumption of nitrite; 1) dissimilatory nitrite reduction under culture conditions including sufficient organic matter as an electron donor and 2) assimilatory nitrite reduction. Since organic matter in the medium used in this experiment was only metabolites produced by AM1P, the possibility of dissimilatory nitrite reduction was low. Assimilatory nitrite reduction may be possible. We confirmed *nirB* and *nirD* gene expression in NB1 (see below). However, nitrite consumption by assimilatory nitrite reduction may be low and negligible to nitrite consumption shown in Fig. 1.

We then examined the growth activity of AM1P at high cell densities (axenic culture vs. co-culture). The axenic culture (AM1P; 1.0×10^5^ cell mL^–1^) and the co-culture suspended in NB1 at different cell densities (AM1P; 1.0×10^5^ cell mL^–1^, NB1; 10^4^–10^7^‍ ‍cells‍ ‍mL^–1^) were cultivated in 2.0‍ ‍mM of inorganic medium. After an incubation for 15 days, the axenic culture and co-culture both completely consumed nitrite (Fig. S2). The co-existence of NB1 did not affect the nitrite consumption rate of AM1P at higher cell densities. Under high cell density conditions with AM1P growth, difficulties were associated with identifying any effect of NB1. Therefore, we considered an acclimation process to be required in order to induce a mutualistic interaction between AM1P and NB1. With reference to a previous study on the interaction between AOB and heterotrophs (Sedlacek *et al.*, 2016), we conducted a pre-incubation to adapt NB1 to co-exist with AM1P in inorganic medium. The axenic culture (AM1P; 5.0×10^5^ cells‍ ‍mL^–1^) and co-culture (AM1P; 5.0×10^5^ cells‍ ‍mL^–1^ and NB1; 5.0×10^4^ cells‍ ‍mL^–1^) were performed using inorganic medium containing 3.6‍ ‍mM nitrite as a pre-cultivation. When nitrite was completely consumed, pre-cultured samples (axenic culture and co-culture) were transferred to fresh inorganic medium containing 3.6‍ ‍mM nitrite. Based on the nitrite concentration and cell density, we measured the specific growth rate, generation time, and growth yield of AM1P (axenic culture vs. co-culture) (Table 1). We found that the growth rate and yield of AM1P slightly increased in the presence of NB1, whereas no significant difference was observed in nitrite consumption between the axenic culture and co-culture at high cell densities. As described above, the nitrite consumption of AM1P at low cell densities was strongly initiated in the presence of NB1. Throughout this physiological characterization, we hypothesized that AM1P may cause a dynamic metabolic shift by co-existing with NB1, even if apparent growth parameters were nearly the same between the axenic culture and co-cultures. To verify this hypothesis, we conducted a comparative transcriptome ana­lysis of the axenic culture and co-culture.

Total RNA was extracted from the axenic culture and co-cultures during logarithmic nitrite consumption (Fig. S3). A total of 113,784,929 mRNA reads were obtained from two axenic culture samples and three co-culture samples. Retrieved transcriptome sequence data have been deposited in the DNA Databank of Japan (DDBJ) database, EMBL Nucleotide Sequence Database, and National Center for Biotechnology Information (NCBI) database under accession numbers SAMD00560594–SAMD00560598 at the BioProject PRJDB14824. All 2,678 predicted protein-coding sequences (CDS) of AM1P (Ishii *et al.*, 2020) were detected (Table S1). Regarding NB1, 5,004 out of 5,011 CDS were mapped (Ide *et al.*, 2019) (Table S2). A significant change was defined as a difference in AM1P gene expression of log_2_ fold-change |1.0| with *P*<0.05 for the co-culture vs. the axenic culture. Among the 107 genes with significant changes in expression, 46 were up-regulated and 61 were down-regulated (Fig. S4). Significant differentially expressed genes were identified based on clusters of orthologous groups. We focused on the energy synthesis and nitrogen metabolism of AM1P in the co-culture (Fig. 2).

The transcript abundance of nitrite oxidoreductase genes (*nxrABC*) (W01_16870, W01_12950, W01_16860, W01_14020, W01_12940, and W01_14030) slightly increased. AM1P grown in the co-culture significantly increased the transcript levels of genes encoding the liposome protein (W01_22860) and cell division protein FtsH (W01_24700). These gene expression profiles showed that the generation time of AM1P was shorter in the co-culture than in the AM1P axenic culture (Table 1). Regarding nitrogen metabolism, the co-culture showed a significant increase in the expression of the assimilatory nitrite reductase *nirB* (W01_03820) and ammonium transporter *amtB* (W01_00340). AM1P may actively produce ammonium to synthesize N-containing compounds, such as amino acids. Amino acids may be released as a byproduct and supplied to NB1 because three marine AOA strains were previously shown to exude dissolved organic matter, including N-containing compounds (Bayer *et al.*, 2019). NB1 in the culture highly expressed the glutamine synthetase *glnA* gene (ACINB_00240) at a high‍ ‍level, similar to that of amino acid transporters (ACINB_20500 and ACINB_30160), which are periplasmic binding proteins. Furthermore, NB1 in the culture highly expressed *nirB* (ACINB_14400), *nirD* (ACINB_14410), D-amino acid dehydrogenase (DAD) (ACINB_23850) and glutamate dehydrogenase (GDH) (ACINB_23490) deaminating glutamate into α-ketoglutaric acid and ammonium. Ammonium excreted by NB1 may be transported to the inner cell of AM1P via its AmtB and reused in AM1P as a nitrogen source. The recycling of nitrogen sources between AM1P and NB1 may be one of the reasons for the stimulated growth of AM1P in the co-culture. This is consistent with our previous findings, which demonstrated that the nitrite oxidation rate of AM1P increased in the presence of ammonium (Ishii *et al.*, 2020).

We previously attempted to purify *Ca.* Nitrotoga-like cells from a cell sorter-based enrichment culture by serial dilutions using 96-well microtiter plates; however, nitrite oxidation was not confirmed even after an incubation for 1 year at all dilution levels (1–100 cells well^–1^). These results imply that *Ca.* Nitrotoga and heterotrophs cooperatively interact with each other in microscale communities (*i.e.*, *Ca.* Nitrotoga aggregates). AM1P grew in 96-well plates inoculated with aggregates containing heterotrophs including *Acidovorax* (Ishii *et al.*, 2020). The beneficial interactions between *Ca.* Nitrotoga and heterotrophs may involve N-containing compounds.

In conclusion, we herein demonstrated that nitrite consumption by the nitrite-oxidizing *Ca.* Nitrotoga strain was promoted in the presence of heterotrophs during the initial incubation. Although the specific growth rate, generation time, and growth yield of the *Ca.* Nitrotoga strain were not significantly different between the axenic culture and co-culture, some genes relevant to nitrogen metabolism (*e.g.*, ammonium transporter) in the *Ca.* Nitrotoga strain were up-regulated at the transcriptomic level in the co-culture.

## Citation

Ide, H., Ishii, K., Takahashi, Y., Fujitani, H., and Tsuneda, S. (2023) Effects of Co-existing Heterotrophs on Physiology of and Nitrogen Metabolism in Autotrophic Nitrite-oxidizing *Candidatus* Nitrotoga. *Microbes Environ ***38**: ME23076.

https://doi.org/10.1264/jsme2.ME23076

## Supplementary Material

Supplementary Material 1

Supplementary Material 2

Supplementary Material 3

Supplementary Material 4

## Figures and Tables

**Fig. 1.**
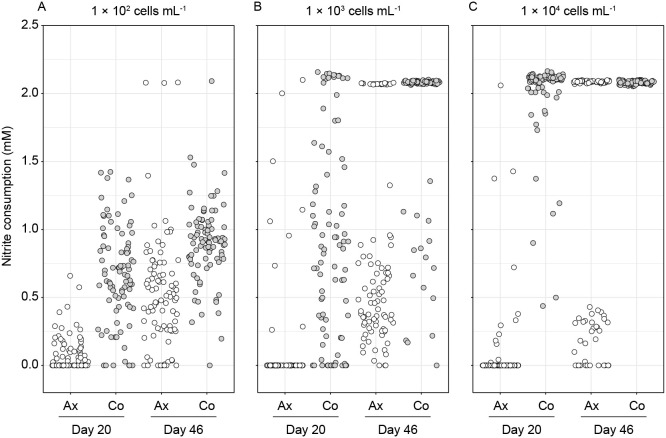
Nitrite consumption in each well of a 96-well plate in the incubation experiment at low cell densities. Nitrite concentrations were measured after an incubation for 20 and 46 days. Each culture condition contains 88 plots equivalent to the number of wells used for incubation. Ax (white plots); axenic culture of *Candidatus* Nitrotoga sp. AM1P. Co (gray plots); co-culture of AM1P and *Acidovorax* sp. NB1. The initial cell concentrations of both strains were adjusted at (A) 1×10^2^, (B) 1×10^3^, or (C) 1×10^4^‍ ‍cells‍ ‍mL^–1^.

**Fig. 2.**
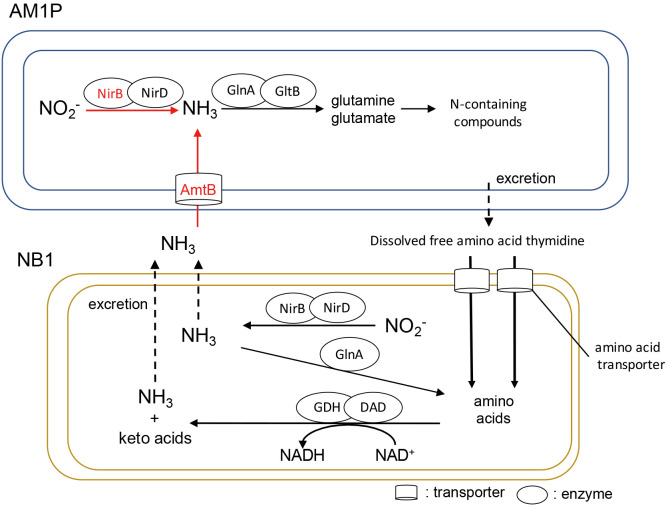
Nitrogen metabolism and regulation of the mutualistic interaction between AM1P and NB1 expected from the transcriptomic ana­lysis. In AM1P cells, black letters; genes with increased transcription, arrows shown with black solid lines; reactions catalyzed by genes with increased transcription, red letters; genes with significantly increased transcription (*P*<0.05), arrows shown with red solid lines; reactions catalyzed by genes with significantly increased transcription, arrows shown with black dashed lines; reactions possibly occurring between AM1P and NB1. In NB1 cells, nitrogen metabolism relevance to the top 200 most highly transcribed genes of NB1 were described. AmtB; ammonia/ammonium transporter (W01_00340), Amino acid transporter (ACINB_20500, ACINB_30160), DAD; D-amino acid dehydrogenase (ACINB_23850, ACINB_26800, ACINB_35240), GDH; glutamate dehydrogenase (ACINB_23490), GlnA; glutamine synthetase (W01_14570 for AM1P; ACINB_00240 for NB1), GltB; glutamate synthetase (W01_13290), NirB; nitrite reductase (W01_03820 for AM1P; ACINB_14400 for NB1), NirD; nitrite reductase (W01_03830 for AM1P; ACINB_14410 for NB1).

**Table 1.** Specific growth rate, generation time, and growth yield of AM1P in an axenic culture (only AM1P) and co-culture (AM1P+NB1)*^a^* at high cell densities and with an acclimation process

	Specific growth rate (h^–1^)	Generation time (h)	Growth yield log_10_ (cells NO_2_^–^ mM^–1^)
AM1P	0.0116 (±0.00578)	73.0 (±27.1)	9.54 (±0.10)
AM1P+NB1	0.0115 (±0.00303)	65.7 (±20.8)	9.65 (±0.14)

*^a^* An exponential growth curve was fit based on the least squares method between the first day when nitrite decreased and the day when nitrite was completely consumed. The specific growth rate, generation time, and growth yield were calculated in the approximated growth curve. Values are averages of biological triplicates. Brackets show the standard deviation (*n*=3).
