# Supplementary material for: Effects of Co-existing Heterotrophs on Physiology of and Nitrogen Metabolism in Autotrophic Nitrite-oxidizing Candidatus Nitrotoga
Source: Microbes Environ. 2023 Dec 9;38(4):ME23076. doi: 10.1264/jsme2.ME23076 (PMC10728634; doi:10.1264/jsme2.ME23076)
Supplement: Supplementary file 1 — Supplementary Material 1 [file 38_23076_s1.pdf]

Supplementary Material

Materials and Methods

Strains and Culture conditions

Ca. Nitrotoga sp. AM1P isolated from eelgrass sediment (Ishii et al., 2020) was adopted as a representative nitrite oxidizer. The AM1P culture in early stationary phase that was incubated for 3 days after nitrite depletion was used for cultivation tests. As a heterotrophic bacterium, *Acidovorax* sp. NB1 isolated from AM1P enrichment (Ide et al., 2019) was used to investigate mutualistic interaction between autotrophic nitrite oxidizer and heterotroph. The NB1 cells stocked in a deep freezer were suspended with nutrient broth (NB) medium (Difco) and incubated at 29°C in a shaking and dark condition for 48 h. After that, the NB1 cells in early stationary phase were obtained. Cells of both strains were centrifuged (2,900 × *g*, 10 min) and filtered with a 0.22 μm pore size filter. The cell pellets were carefully washed with 40 mL inorganic medium. The centrifugation and washing were conducted twice to prevent contamination of the original medium, referring a previous study (Murakami et al., 2021).

The inorganic medium has the following composition diluted in one liter of water: K_2HPO_4 (25.4 mg), $MgSO_4 \cdot 7H_2O$ (40.6 mg), $CaCl_2 \cdot 2H_2O$ (6.6 mg), $FeSO_4 \cdot 7H_2O$ (3.2 mg), $NaHCO_3$ (200 mg), $MnSO_4 \cdot 5H_2O$ (54.2 μg), H_3BO_3 (49.4 μg), $ZnSO_4$ (43.1 μg), $Na_2Mo_4O_4$ (27.6 μg), $CuSO_4 \cdot 5H_2O$ (25 μg) (Ishii et al., 2020). The initial pH was adjusted to 7.8–8.2. The medium was used for cultivation after autoclaving and filtration (pore size, 0.22 μm). For contamination test, NB medium was used.

Nitrite consumption of AM1P in low cell density (axenic culture vs coculture)

AM1P and AM1P + NB1 cultures were incubated in 96-well microtiter plates in the dark and static conditions. Cell suspensions in 150 μL of nitrite (2.1 mM) inorganic medium were inoculated into each well. In both axenic culture (only AM1P cells) and coculture (AM1P + NB1 cells), cell densities of AM1P were prepared with 10^2-10^4 cells mL^{-1}. Cell densities of NB1 were equivalent to those of AM1P

in coculture. On days 20 and 46, nitrite concentrations were measured in each well. Cell growth of AM1P was observed with fluorescence *in situ* hybridization (FISH) microscopic analysis.

Measurement of nitrite and nitrate concentrations

Nitrite concentration was determined using Griess reagent (Griess-Romijn van Eck, 1966). Absorbance at a wavelength of 550 nm was measured using a Powerscan HT multiplate reader (DS Pharma Biomedical, Osaka, Japan). Additionally, nitrite and nitrate concentrations were measured using an IC-2010 ion chromatography system (Tosoh Co. Tokyo, Japan).

Growth activity of AM1P, Culture for RNA sequencing (axenic culture vs coculture)

In the first trial, axenic culture (AM1P; 1.0×10^5 cell mL^{-1}) and cocultures suspended in different NB1 cell densities (AM1P; 1.0×10^5 cell mL^{-1}, NB1; 10^4 – 10^7 cells mL^{-1}) were cultivated in the inorganic medium containing 2.0 mM nitrite for approximately two weeks. In the second trial, axenic culture (AM1P; 5.0×10^5 cells mL^{-1}) and coculture (AM1P; 5.0×10^5 cells mL^{-1} and NB1; 5.0×10^4 cells mL^{-1}) were cultivated in the inorganic medium containing 3.6 mM nitrite as pre-cultivation at 16°C in the static and dark condition. When the nitrite was completely consumed, the pre-cultured samples were transferred to the fresh inorganic medium containing 3.6 mM nitrite, in which AM1P cells were prepared as 5.0×10^5 cells mL^{-1}. Nitrite concentration and cell density were measured every 2 or 3 days with ion chromatography and microscopy, respectively.

For RNA sequencing, AM1P cultures were incubated under starvation condition during three days. For pre-cultivation, axenic culture (AM1P; 5.0×10^5 cells mL^{-1}) and coculture (AM1P; 5.0×10^5 cells mL^{-1} and NB1; 5.0×10^5 cells mL^{-1}) were cultivated in the inorganic medium containing 4 mM nitrite as 1.3 L volume at 16°C in the static and dark condition. When the nitrite was completely consumed, a part of the pre-cultured samples was transferred to the fresh inorganic medium containing 4 mM nitrite for

subsequent cultivation. Three replicate samples of axenic culture (only AM1P) and coculture (AM1P + NB1) were collected in the exponential phase in less than 3 mM of nitrite concentration on days 4 and 3, respectively (Fig. S3). The samples were harvested by filtering (pore size 0.22 μm) at 4°C. The pellets on the filters were suspended in the fresh inorganic medium. The suspensions were centrifuged (2,900 × *g*, 10 min) to remove the supernatant. Finally, the pellets were preserved at −80°C.

Microscopic observation and estimation of cell density

Cell densities of AM1P and NB1 were estimated with direct counting under a fluorescent microscopy (Axioskop 2 plus: Carl Zeiss, Oberkochen, Germany). Cells of AM1P and NB1 were specifically detected based on FISH method and nucleotide acid staining. FISH was conducted according to a previous protocol (Amann et al., 1990). As a *Ca.* Nitrotoga-specific FISH probe, NTG840 (5' CTA AGG AAG TCT CCT CCC 3') was used (Alawi et al., 2007). As nucleotide acid staining, SYTOX green (Invitrogen) was used.

RNA extraction and transcriptome sequencing

Total RNA of the cell pellets preserved at −80°C was extracted using RNeasy mini kit (Qiagen). Concentration and purity of extract RNA were checked using a NanoDrop ND-1000 spectrophotometer (Thermo Fisher Scientific, Inc., Wilmington, DE, US) and the Qubit Fluorometer with Qubit RNA HS assay kit (Thermo Fisher Scientific, Inc., Wilmington, DE, US), respectively. To prevent DNA contamination, DNase treatment was conducted using RNA Clean & Concentrator-5, DNase included (Zymo Research R1014); for DNase treatment. Ribosomal RNA was removed using QIAseq FastSelect – 5S/16S/23S Kit (QIAGEN #335927). Sequencing libraries were constructed using NEBNext Ultra II Directional RNA Library Prep Kit for Illumina (New England BioLabs #7760). The 150-bp pair-end libraries were sequenced with Illumina Novaseq. DNase treatment, removal of rRNA, library preparation, and sequencing were conducted at GENEWIZ (https://www.genewiz.com).

Transcriptome analysis

The quality of the reads was checked using FastQC (v0.11.9). The trimming of the adapter, low quality reads (quality score < 30), and short reads (< 36 bp) was conducted using trimmomatic (version 0.39). The paired-end transcriptome sequence reads were aligned to open reading frames (ORFs) deposited at Genbank for *Ca.* Nitrotoga sp. AM1P (GenBank: AP019547.1) reference genome and *Acidovorax* sp. NB1 (GenBank: BJHU00000000) reference genome using Bowtie2 (version 2.4.1). Based on the mapping files, read counts including CDS annotation region of AM1P and NB1 were generated using featureCounts. The mapped read count was normalized as Relative log expression (RLE) to compare between samples using DESeq2. Based on student t-test, $\log_2$ fold change between AM1P genes under two culture conditions was calculated. For NB1, normalization based on transcript per million (TPM) was conducted using the following formula.

$$T_t = \frac{Y_t}{L_t} 10^3 \qquad \text{-(1)}$$

$$TPM_t = T_t \frac{1}{\sum_t T_t} 10^6 \qquad \text{-(2)}$$

Y_t : read count mapped to transcript t; L_t: length of transcript t (bp); T_t: read number per 1,000 bp of transcript t; TPM_t: Gene expression level of transcript t per total transcript 1 million.

References

Alawi, M., Lipski, A., Sanders, T., Eva-Maria-Pfeiffer, and Spieck, E. (2007) Cultivation of a novel cold-adapted nitrite oxidizing betaproteobacterium from the Siberian Arctic. *ISME J* **1**: 256-264.

Amann, R., Krumholz, L., and Stahl, D. (1990) Fluorescent-oligonucleotide probing of whole cells for determinative, phylogenetic, and environmental-studies in microbiology. *J Bacteriol* **172**: 762-770.

Griess-Romijn van Eck, E. (1966) Physiological and Chemical Tests for Drinking Water. NEN 1056, IV-2. Rijswijk, The Netherlands: Nederlands Normalisatie Instituut.

Ide, H., Ishii, K., Fujitani, H., and Tsuneda, S. (2019) Draft Genome Sequence of *Acidovorax* sp. Strain NB1, Isolated from a Nitrite-Oxidizing Enrichment Culture. *Microbiol Resour Announc* **8**.

Ishii, K., Fujitani, H., Sekiguchi, Y., and Tsuneda, S. (2020) Physiological and genomic characterization of a new '*Candidatus* Nitrotoga' isolate. *Environ Microbiol* **22**: 2365-2382.

Murakami, C., Machida, K., Nakao, Y., Kindaichi, T., Ohashi, A., and Aoi, Y. (2022) Mutualistic relationship between *Nitrospira* and concomitant heterotrophs. *Environ Microbiol Rep* **14**: 130-137.

Figure legends

Fig. S1

Number of wells showing consumption of more than 0.21 mM nitrite.

Fig. S2

Nitrite consumption and nitrate production of axenic culture (only AM1P) and cocultures (AM1P + NB1) suspended in different cell densities of NB1 (10^4 – 10^7 cells mL^{-1}). Open circle shows control without NB1. Open square shows NB1 in 10^7 cells mL^{-1}. Open diamond shows NB1 in 10^6 cells mL^{-1}. Open triangle shows NB1 in 10^5 cells mL^{-1}. Cross mark shows NB1 in 10^4 cells mL^{-1}. Error bar shows biological triplicates.

Fig. S3

Nitrite consumption of axenic culture (only AM1P) and coculture (AM1P + NB1) prepared for RNA sequencing. Open circle shows axenic culture. Closed circle shows coculture. For RNA extraction, samples of axenic culture and coculture were collected on days 4 and 3, respectively. The collected days are shown with arrows. Error bar shows biological triplicates.

Fig. S4

Volcano plot of *Ca.* Nitrotoga sp. AM1P showing the relationship between different expression change ratio (log2 fold change) and *p*adj value of different expression (-log10 adjusted *p* value). Red plots show significantly upregulated genes ($log_2FC > 1.0$, -log10 (*p*adj) > 0.05). Blue plots show significantly downregulated genes ($log_2FC < -1.0$, -log10 (*p*adj) > 0.05). Black plots show no different expression change genes. Dashed line shows $|log_2FC| > 1.0$, -log10 (*p*adj) > 0.05.

Table S1

Gene expression levels of *Ca.* Nitrotoga sp. AM1P genes in coculture with *Acidovorax* sp. NB1 compared to axenic culture.

Table S2

Read count of *Acidovorax* sp. NB1 in coculture with *Ca.* Nitrotoga sp. AM1P.

Table S3

Top 200 most highly transcribed genes of *Acidovorax* sp. NB1.

Fig. S1

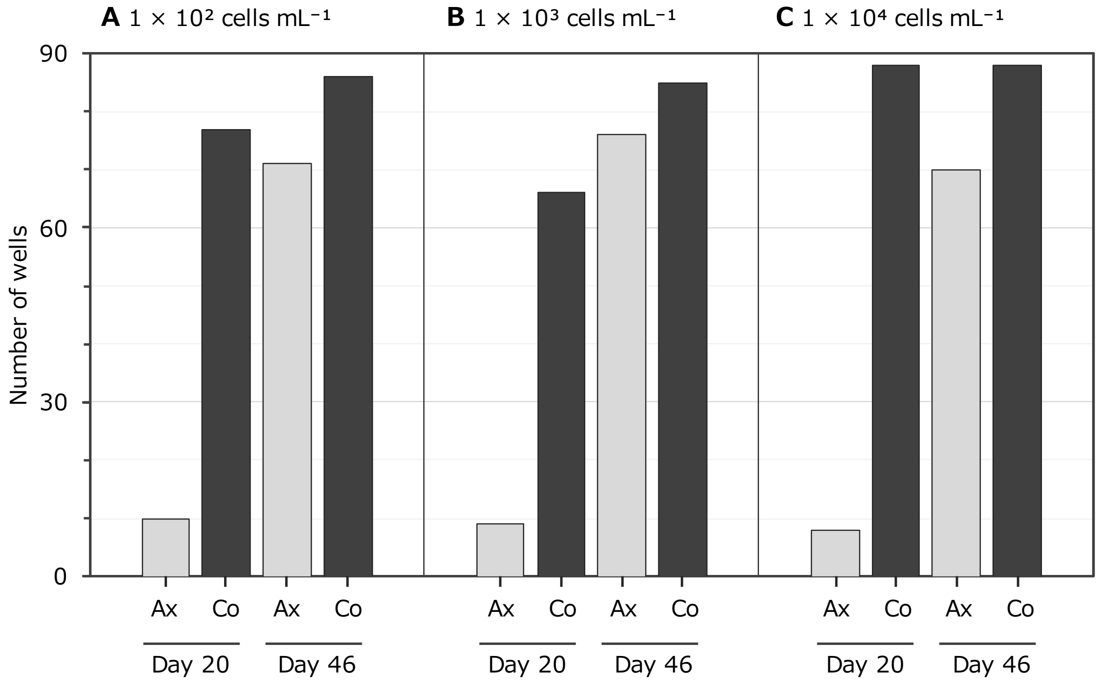
A 1 × 10² cells mL⁻¹
B 1 × 10³ cells mL⁻¹
C 1 × 10⁴ cells mL⁻¹
Number of wells
0
30
60
90
Ax
Co
Day 20
Day 46

Fig. S2

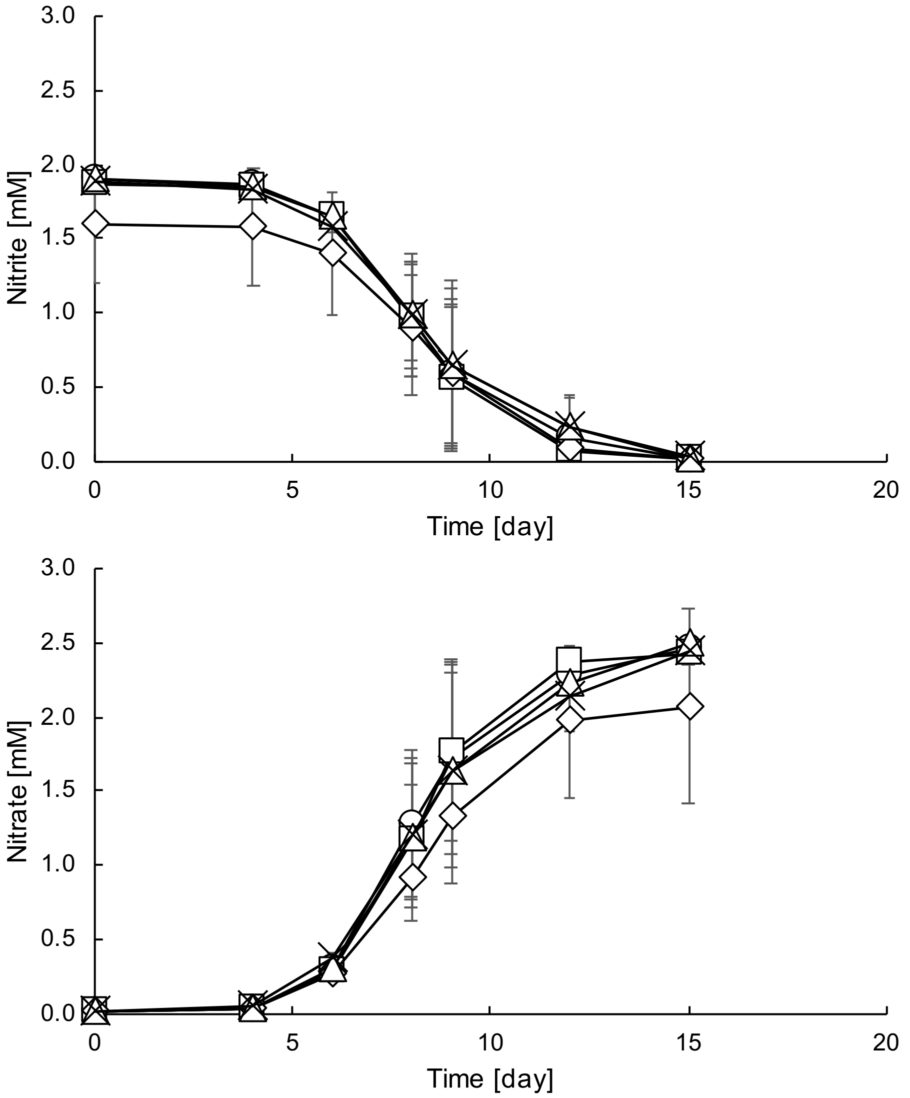
3.0
2.5
2.0
1.5
1.0
0.5
0.0
Nitrite [mM]
0
5
10
15
20
Time [day]
3.0
2.5
2.0
1.5
1.0
0.5
0.0
Nitrate [mM]
0
5
10
15
20
Time [day]

Fig. S3

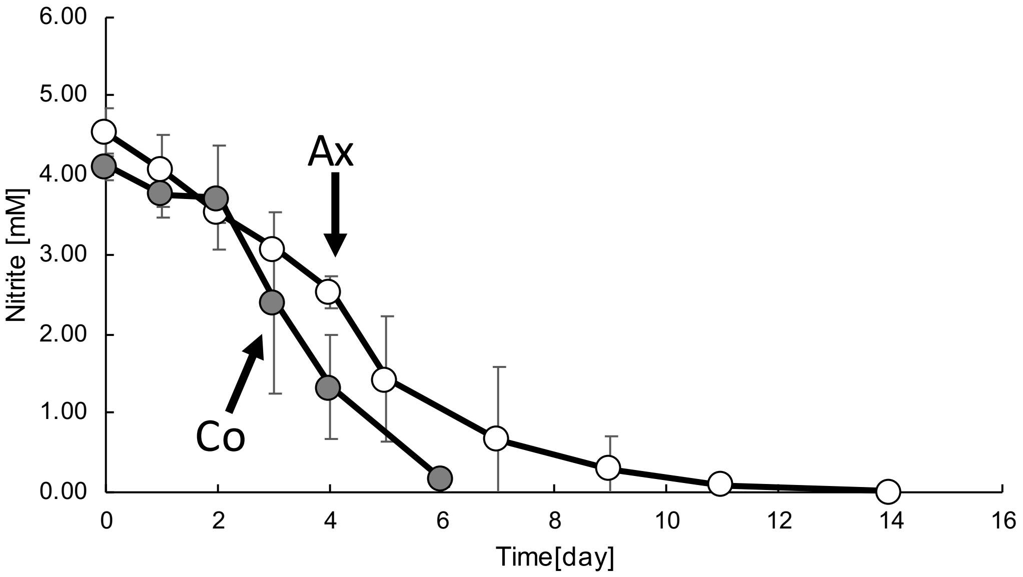

6.00
5.00
4.00
3.00
2.00
1.00
0.00
Nitrite [mM]
0
2
4
6
8
10
12
14
16
Time[day]
Ax
Co

Fig. S4

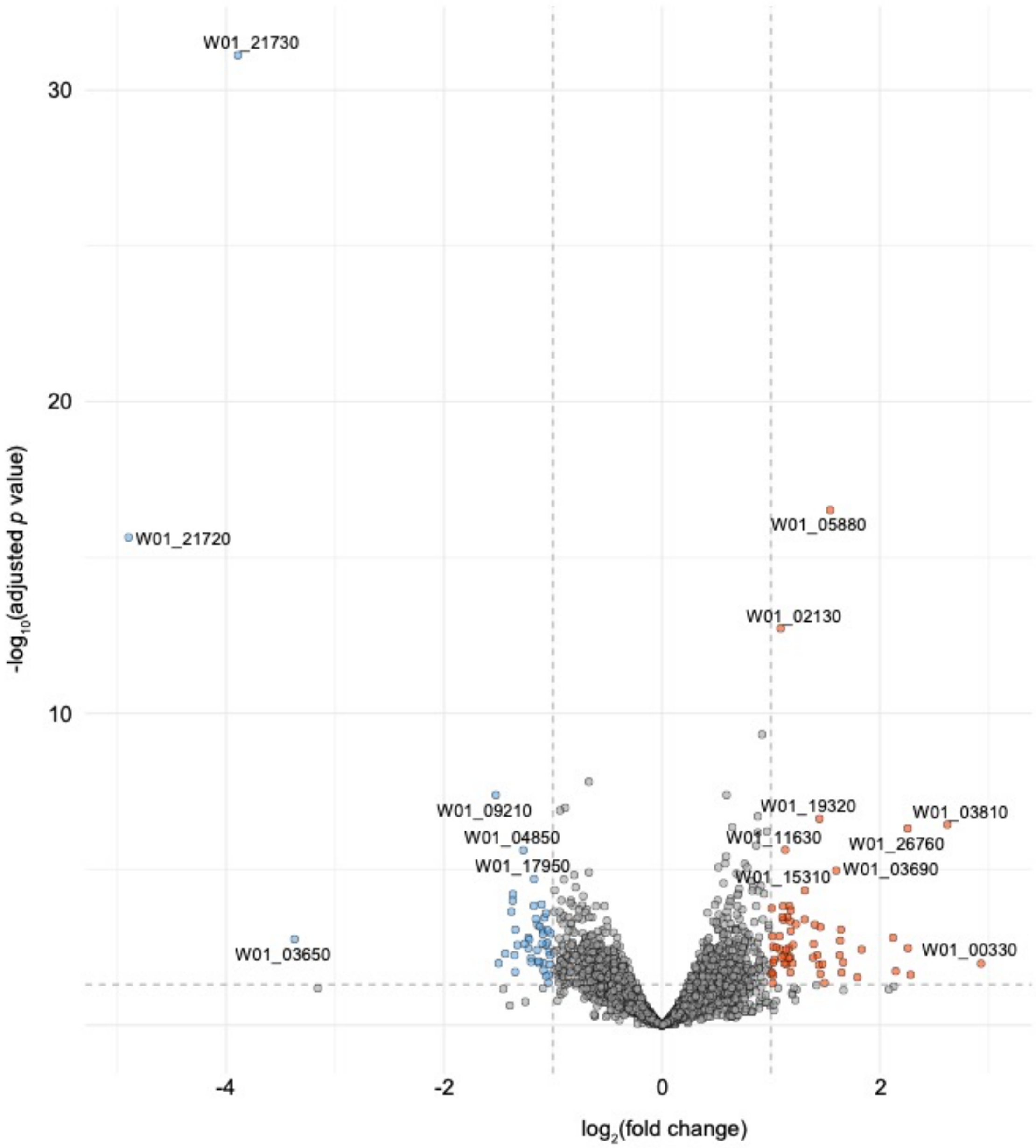
W01_21730
W01_21720
W01_05880
W01_02130
W01_09210
W01_04850
W01_17950
W01_19320
W01_11630
W01_15310
W01_03810
W01_26760
W01_03690
W01_03650
W01_00330
30
20
10
-4
-2
0
2
-log10(adjusted p value)
log2(fold change)
